# The Chemopreventive Phytochemical Moringin Isolated from *Moringa oleifera* Seeds Inhibits JAK/STAT Signaling

**DOI:** 10.1371/journal.pone.0157430

**Published:** 2016-06-15

**Authors:** Carina Michl, Fabio Vivarelli, Julia Weigl, Gina Rosalinda De Nicola, Donatella Canistro, Moreno Paolini, Renato Iori, Anne Rascle

**Affiliations:** 1 Stat5 Signaling Research Group, Institute of Immunology, University of Regensburg, Regensburg, Germany; 2 Molecular toxicology unit, Department of Pharmacy and Biotechnology, University of Bologna, Bologna, Italy; 3 Consiglio per la ricerca in agricoltura e l'analisi dell'economia agraria, Centro di ricerca per le colture industriali (CREA-CIN), Bologna, Italy; University of St Andrews, UNITED KINGDOM

## Abstract

Sulforaphane (SFN) and moringin (GMG-ITC) are edible isothiocyanates present as glucosinolate precursors in cruciferous vegetables and in the plant *Moringa oleifera* respectively, and recognized for their chemopreventive and medicinal properties. In contrast to the well-studied SFN, little is known about the molecular pathways targeted by GMG-ITC. We investigated the ability of GMG-ITC to inhibit essential signaling pathways that are frequently upregulated in cancer and immune disorders, such as JAK/STAT and NF-κB. We report for the first time that, similarly to SFN, GMG-ITC in the nanomolar range suppresses IL-3-induced expression of STAT5 target genes. GMG-ITC, like SFN, does not inhibit STAT5 phosphorylation, suggesting a downstream inhibitory event. Interestingly, treatment with GMG-ITC or SFN had a limited inhibitory effect on IFNα-induced STAT1 and STAT2 activity, indicating that both isothiocyanates differentially target JAK/STAT signaling pathways. Furthermore, we showed that GMG-ITC in the micromolar range is a more potent inhibitor of TNF-induced NF-κB activity than SFN. Finally, using a cellular system mimicking constitutive active STAT5-induced cell transformation, we demonstrated that SFN can reverse the survival and growth advantage mediated by oncogenic STAT5 and triggers cell death, therefore providing experimental evidence of a cancer chemopreventive activity of SFN. This work thus identified STAT5, and to a lesser extent STAT1/STAT2, as novel targets of moringin. It also contributes to a better understanding of the biological activities of the dietary isothiocyanates GMG-ITC and SFN and further supports their apparent beneficial role in the prevention of chronic illnesses such as cancer, inflammatory diseases and immune disorders.

## Introduction

Chronic pathological conditions such as cancer, inflammatory diseases and immune disorders figure among the leading causes of mortality worldwide [1,2]. Dietary chemoprevention received increased attention over the past few years as an approach to lower cancer incidence and mortality [3,4]. The isothiocyanate (ITC) sulforaphane (1-isothiocyanato-4-(methylsulfinyl)-butane, thereafter referred to as SFN) is considered an ideal chemopreventive agent, due to its abundance in easily accessible cruciferous vegetables, its excellent bioavailability, its ability to target multiple pathways and its low toxicity [5–9]. Part of the pleiotropic biological activity of SFN depends on its electrophilic nature, resulting in the direct and/or indirect covalent modification of molecules, including proteins, thus altering their function [10]. Edible electrophiles such as SFN were shown to exert anti-inflammatory activities for instance through the activation of the Nrf2-dependent pathway and the inhibition of NF-κB signaling [10–19].

We also reported recently that SFN inhibits STAT5-mediated transcription [20]. STAT5 (Signal Transducer and Activator of transcription 5) belongs to a family of transcription factors (STAT1-6) present mainly in an inactive form in the cytoplasm of numerous cell types and which are activated by phosphorylation in response to specific cytokines, hormones and growth factors [21–27]. STAT5 is essential for the control of cell proliferation and survival, via the regulation of genes such as *c-Myc*, *Pim-1*, *Bcl-x*, *Osm* or *Cis* [22,28,29]. STAT5 also contributes to immune cell differentiation and function [27]. Its activity is normally tightly controlled, and is hence frequently found dysregulated in malignancies and immune disorders [22,27,30–32]. Notably, STAT5 is commonly constitutively activated in cancer [30,31,33], and is therefore a target of choice for cancer therapy and prevention [33–37]. A number of small-molecules inhibitors of the STAT5 signaling pathway have been reported. Most of them, whether natural or synthetic, target JAK2, a tyrosine kinase responsible for STAT5 phosphorylation [34–46]. Inhibitors targeting STAT5 protein itself and its transcriptional activity have also been described [20,47–53]. We showed that deacetylase inhibitors and SFN inhibit STAT5 activity at the transcriptional level, at a step following the binding of STAT5 to DNA [20,53,54]. Other STAT family members, in particular STAT1 and STAT2, exert essential immune-modulation functions, in particular in response to infection. They are activated by type I interferons (IFNα/β) via the JAK1 and TYK2 tyrosine kinases. Phosphorylated STAT1 and STAT2 associate with IRF9 to form the ISGF3 complex, which translocates into the nucleus and activates the transcription of downstream IFN-stimulated genes or ISGs [55,56]. Alteration of IFN signaling is linked to immunodeficiencies, auto-immune diseases and cancer [21,27]. Altogether, it has been recognized that adequate regulation of the JAK/STAT and NF-κB pathways is crucial for the maintenance of proper immune functions and of controlled cell proliferation and survival, and hence afford prevention of chronic pathologies such as immune disorders, inflammatory diseases and cancer.

**Fig 1:**
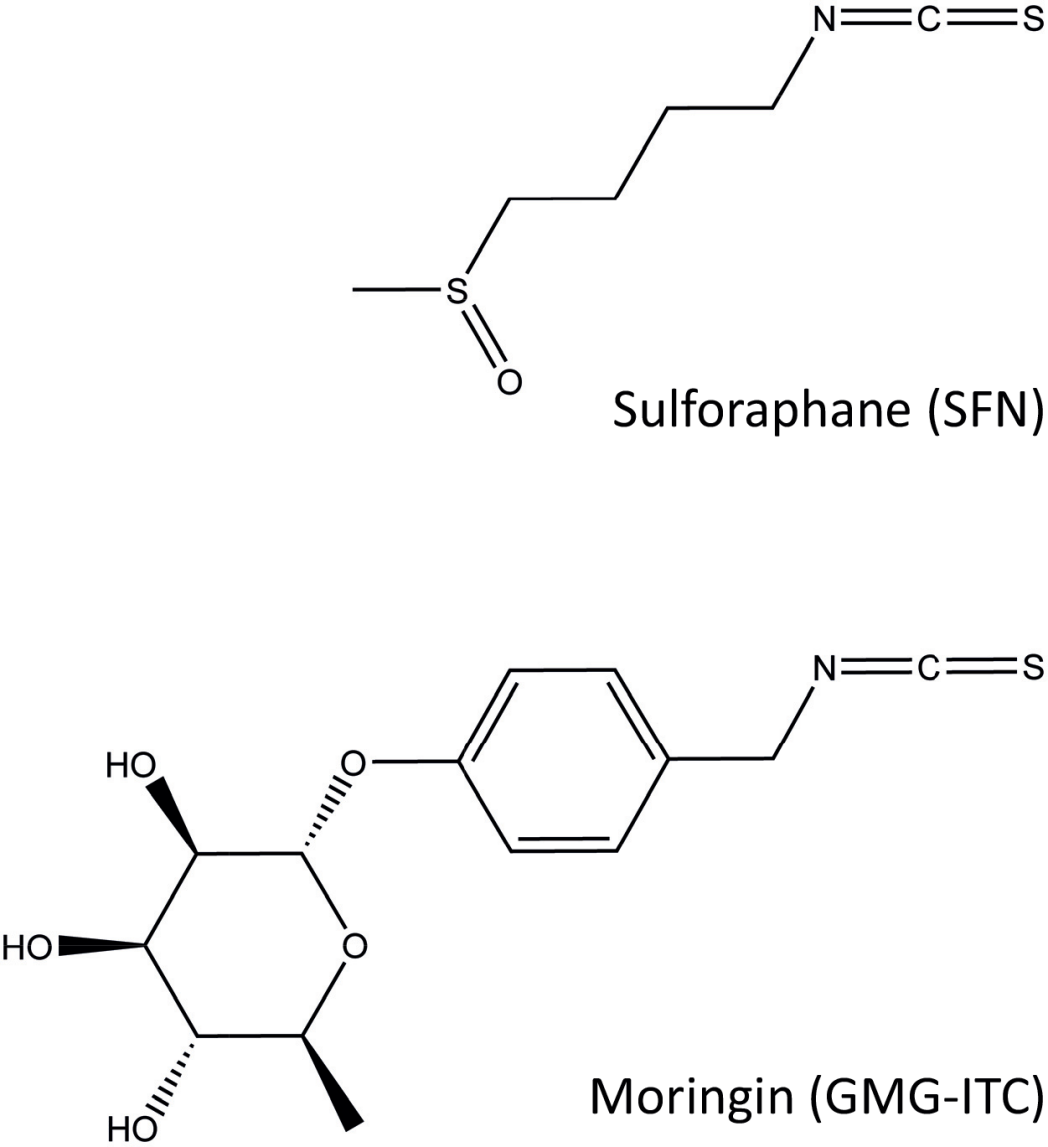
Structure of the natural isothiocyanates sulforaphane (SFN) and moringin (GMG-ITC). Sulforaphane (SFN; 1-isothiocyanato-4-(methylsulfinyl)-butane, also called glucoraphanin isothiocyanate or GRA-ITC) and moringin (4-(α-L-rhamnopyranosyloxy)-benzyl isothiocyanate, also referred to as glucomoringin isothiocyanate or GMG-ITC) are produced by myrosinase-catalyzed hydrolysis of their glucosinolate precursors, glucoraphanin (GRA) and glucomoringin (GMG) respectively [59].

Beside SFN, another edible ITC from the plant *Moringa oleifera*, 4-(α-L-rhamnopyranosyloxy)-benzyl ITC, also called moringin (thereafter referred to as GMG-ITC), recently attracted the attention of scientists for its chemopreventive activity. GMG-ITC is produced by myrosinase-catalyzed hydrolysis of glucomoringin (GMG) [57–59]. *Moringa oleifera* is renowned for its medicinal virtues and nutritional value. All parts of the plant (leaves, seeds, roots, bark, flowers) are consumed or used in preparations for the treatment of various ailments, including infections, inflammation, hypertension and diabetes [60]. GMG-ITC has been recently characterized [58] and was shown to exert anti-inflammatory activities both *in vitro* and *in vivo*, notably through the inhibition of the NF-κB pathway [57,61–63]. Thus, like other ITCs and food electrophiles [10,64], GMG-ITC shares with SFN the ability to inhibit NF-κB signaling. Interestingly, GMG-ITC exhibited anti-tumoral activity in a mouse model of multiple myeloma, which is considered to be dependent on NF-κB [57]. On the other hand, moringin was recently described as a potent inducer of apoptosis in a human malignant astrocytoma cell line [65]. Beside these scarce observations, very little is known about the effect of GMG-ITC on other signaling pathways, such as JAK/STAT. SFN and GMG-ITC present distinct structures (Fig 1), raising the possibility that they might possess non-redundant biological activities.

In this study, we investigated the potential inhibitory activity of GMG-ITC on a number of signaling pathways often deregulated in chronic disorders (STAT5, STAT1, STAT2, NF-κB), using the well-characterized SFN as a reference compound. We show—to our knowledge for the first time—that GMG-ITC, similarly to SFN, strongly inhibits STAT5-mediated transcription without altering STAT5 activation by phosphorylation. Whereas both ITCs exerted a limited inhibitory activity toward STAT1/STAT2-mediated transcription, we confirmed that they are potent inhibitors of NF-κB signaling, GMG-ITC being more efficient than SFN. Altogether, our data identified STAT5, NF-κB and, to a lesser extent, STAT1/STAT2 as molecular targets of GMG-ITC and SFN, therefore further supporting the proposed role of these natural ITCs as valuable dietary chemopreventive agents.

## Materials and Methods

### Chemicals

Dimethyl sulfoxide (DMSO) and curcumin were purchased from SIGMA (D-2650 and 08511 respectively). R,S-Sulforaphane (SFN) was from LKT Laboratories (S8044). Moringin (GMG-ITC) was produced by myrosinase-catalyzed hydrolysis of glucomoringin isolated from *Moringa oleifera* Lam. (fam. Moringaceae) seeds and purified by HPLC (>99% purity), as previously described [57,58]. Compounds were dissolved at 100 mM in DMSO (vehicle). DMSO final concentration was adjusted in all experiments to 0.01%, with the exception of the gene expression analysis in TNF-stimulated HeLa cells (0.05% DMSO final) and of the WST-1 assays (0.1% DMSO final).

### Cell lines and drug treatments

All cell lines were cultivated at 37°C under 5% CO_2_ in a humidified incubator. HeLa cells (a kind gift from Daniela Männel, Institute of Immunology, University of Regensburg, Germany; [66]) were grown in DMEM (Gibco 31966–021) supplemented with 10% heat-inactivated fetal calf serum (FCS; PAN-Biotech) and penicillin/streptomycin (100 U/mL penicillin, 100 μg/mL streptomycin; PAN-Biotech). The interleukin-3 (IL-3)-dependent mouse pro-B cell line Ba/F3 (a kind gift from Jacqueline Marvel, International Center for Infectiology Research, Lyon, France; [67]) was grown in RPMI 1640 (PAN-Biotech P04-16500) supplemented with 10% heat-inactivated FCS, penicillin/streptomycin and 2 ng/mL rmIL-3 (ImmunoTools). The Ba/F3-tet-on-1*6 cell line (clone D4.1) conditionally expressing FLAG-tagged constitutively active mouse STAT5A-1*6 has been described [54]. Non-induced Ba/F3-tet-on-1*6 cells were grown in RPMI 1640 supplemented with 10% heat-inactivated FCS, penicillin/streptomycin, 600 μg/mL G418 (SIGMA A-1720), 800 μg/mL hygromycin B (PAN-Biotech) and 2 ng/mL IL-3. Induction of STAT5A-1*6 expression was performed by growing IL-3-withdrawn Ba/F3-tet-on-1*6 cells in RPMI 1640 supplemented with 10% heat-inactivated FCS, penicillin/streptomycin and either 1 μg/mL (first 24 hours) or 100 ng/mL (following 8–11 days) doxycycline (SIGMA D-9891). To achieve a constant concentration of doxycycline over time, given its half-life of 24 hours, fresh doxycycline was added daily to the culture at 50 ng/mL, or provided at 100 ng/mL when medium was changed. Treatment of Ba/F3-tet-on-1*6 cells with SFN was initiated at the same time as doxycycline. Considering its expected instability in culture medium, SFN was added fresh daily. For Western Blot analysis, induced Ba/F3-cells were harvested ~4 hours after the daily addition of doxycycline and SFN.

For drug treatments in RT-qPCR and Western blot assays, HeLa and Ba/F3 cells were incubated with 0.4–10 μM SFN or GMG-ITC, 10–50 μM curcumin, or with 0.01–0.05% DMSO (vehicle), as indicated, for 30 minutes prior to cytokine stimulation. DMSO was adjusted to either 0.01% or 0.05% final, as specified, in all conditions. IFNα stimulation of HeLa cells was performed by incubating cells with 3 ng/mL rhIFNα2a (ImmunoTools) for either 30 minutes (Western blot analysis of STAT1 and STAT2 phosphorylation) or 4 hours (gene expression analysis by RT-qPCR). TNF stimulation of HeLa cells was carried out for 1 hour in the presence of 30 ng/mL TNF (a kind gift from Daniela Männel, Institute of Immunology, University of Regensburg, Germany). For IL-3 stimulation of Ba/F3 cells, cells were washed twice in RPMI 1640 and rested in RPMI 1640 supplemented with 10% heat-inactivated FCS and penicillin/streptomycin for 6 hours before addition of 5 ng/mL IL-3 for either 5 minutes (Western blot analysis of STAT5 phosphorylation) or 30 minutes (gene expression analysis by RT-qPCR).

### Gene expression analysis by quantitative RT-PCR

Following cell harvest, Ba/F3 and HeLa cells were lysed in iScript RT-qPCR sample preparation reagent (170–8899, Bio-Rad Laboratories) at a concentration of 4 x 10^5^ and 1 x 10^5^ cells/mL respectively, and 1 μL was used for cDNA synthesis using iScript cDNA Synthesis kit (170–8891, Bio-Rad Laboratories) following the manufacturer's instructions. Quantitative PCR was performed on a RotorGene Q (Qiagen) as previously described [54], using 0.4 μL (Ba/F3) or 0.8 μL (HeLa) cDNA template per reaction. Data were normalized to either mouse S9 ribosomal mRNA (Ba/F3) or human Lamin A/C (LMNA) mRNA (HeLa), and expressed as relative mRNA levels, like previously reported [20,29,46,53,54,68,69]. Mouse- and human-specific quantitative PCR primers have been described [53,54,69]. Data are mean ± SD of the quantitative PCR performed in either duplicate or triplicate and are representative of at least three independent experiments. Raw data (CT values) are available in the S1 File.

### Cytotoxicity assays

WST-1 assays (11 644 807 001, Roche) were performed to measure changes in mitochondrial dehydrogenase activity as previously described [20,46], on 0.75 x 10^5^ IL-3-growing Ba/F3 cells and 0.6 x 10^4^ HeLa cells pre-treated 30 minutes with 0.1–100 μM SFN, 0.02–20 μM GMG-ITC, or with 0.1% DMSO (vehicle). DMSO concentration was adjusted to 0.1% in all conditions. Duration of incubation with the WST-1 reagent was 2 hours for Ba/F3 cells and 4 hours for HeLa cells, to be comparable to the duration of drug treatment in the gene expression analysis experiments. Data are expressed as a percentage of cytotoxicity relative to vehicle. Each WST-1 assay was performed in triplicate and repeated at least three times for each cell line. Results of one representative experiment are shown. Raw data (OD values 450/620 nm) are available in the S1 File.

### Cell viability assays

The number of living and dead cells was evaluated by Trypan Blue exclusion following SFN or GMG-ITC treatment, as previously described [20,46]. Viable cell density (number of living cells/mL) reflects cell proliferation and survival. Cell death is expressed as the percentage of dead cells. Data shown are representative of at least three independent experiments.

### Protein analysis by Western blot

Whole-cell Brij protein lysis and Western-blot analyses were performed as described [46,54]. Antibodies used for the detection of the respective proteins and their dilutions were: FLAG (M2, SIGMA F-1804; mouse monoclonal; 1:500), pSTAT1 (sc-135648, Santa-Cruz Biotechnology; rabbit polyclonal; 1:1000), pSTAT2 (sc-21689-R, Santa-Cruz Biotechnology; rabbit polyclonal; 1:1000), STAT1 (C-terminus, S21120, BD Transduction Laboratories; mouse monoclonal; 1:1000), STAT2 (C-20, sc-476, Santa-Cruz Biotechnology; rabbit polyclonal; 1:1000), pSTAT5 (#9351, Cell Signaling Technology; rabbit polyclonal; 1:1000), STAT5A (L-20, sc-1081, Santa-Cruz Biotechnology; rabbit polyclonal; 1:1000), STAT5A+B (C-17, sc-835, Santa-Cruz Biotechnology; rabbit polyclonal; 1:1000), α-tubulin (DM1A, sc-32293, Santa-Cruz Biotechnology; mouse monoclonal; 1:200), Anti-Rabbit IgG-Peroxidase (SIGMA A-0545; 1:10,000), Anti-Mouse IgG-Peroxidase (SIGMA A-8924; 1:10,000). Apparent molecular weight of detected proteins was as predicted by the antibody manufacturers. Immunoblots shown are representative of at least two independent experiments. Uncropped original blots are available in the S1 File.

## Results

### Moringin inhibits IL-3-mediated expression of STAT5 target genes

**Fig 2:**
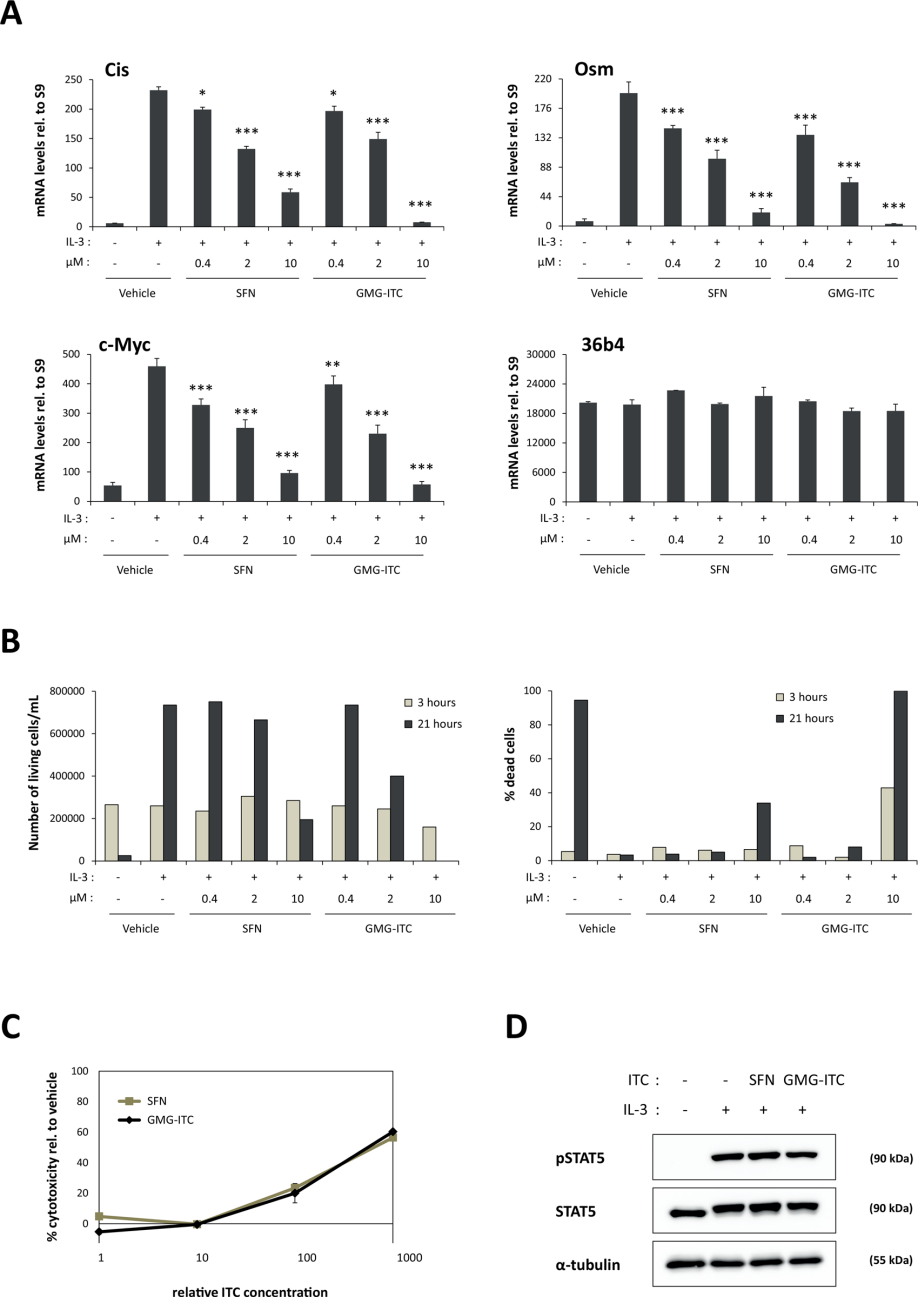
SFN and GMG-ITC inhibit IL-3-mediated induction of STAT5 target genes in a dose-dependent manner in Ba/F3 cells. **(A)** Rested Ba/F3 cells were pre-treated 30 minutes with 0.01% DMSO (vehicle) or with 0.4, 2 and 10 μM SFN or GMG-ITC, and further stimulated 30 minutes with IL-3. Expression of STAT5 target genes (*Cis*, *Osm*, *c-Myc*) and of the housekeeping gene *36b4* was analyzed by quantitative RT-PCR. One-way ANOVA with Dunnett’s post test was used to assess differences between isothiocyanate-treated conditions vs. the vehicle-treated IL-3-stimulated control; *P<0.05, **P<0.01, ***P<0.001. **(B)** Living and dead cells from experiment shown in panel (A) were quantified by trypan blue exclusion 3 hours and 21 hours after beginning of isothiocyanate treatment. Data are expressed as living cell density (left graph) and as percentage of dead cells (right graph). **(C)** GMG-ITC exhibits a higher toxicity than SFN in Ba/F3 cells. Ba/F3 cells were pre-treated 30 minutes with 0.1, 1, 10 and 100 μM SFN or with 0.02, 0.2, 2 and 20 μM GMG-ITC (0.1% DMSO in all conditions) prior to addition of the WST-1 reagent. Assay was conducted for 2 hours, as described in Materials and Methods, and cell toxicity was expressed as a percentage of the vehicle control. Note that the x-axis indicates relative isothiocyanate (ITC) concentrations, with 1 corresponding to 0.1 μM SFN and 0.02 μM GMG-ITC. **(D)** Like SFN, GMG-ITC does not affect IL-3-induced phosphorylation of STAT5. Rested Ba/F3 cells were pre-treated 30 minutes with 10 μM SFN, 2 μM GMG-ITC or 0.01% DMSO as vehicle control (-) and further stimulated 5 minutes with IL-3. Whole-cell protein lysates were analysed by Western blot using antibodies directed against phosphorylated STAT5 (pSTAT5), STAT5A+B (STAT5) and α-tubulin as a loading control. ITC, isothiocyanate.

Ba/F3 are IL-3-dependent mouse pro-B cells commonly used to study STAT5 signaling [20,29,53,54,67]. We showed before that treatment of Ba/F3 cells with the ITC sulforaphane (SFN) inhibits IL-3-induced expression of STAT5 target genes [20]. To investigate the potential inhibitory activity of moringin (GMG-ITC) on STAT5 activity, Ba/F3 cells were treated for 30 minutes with 0.4–10 μM SFN or GMG-ITC, followed by stimulation with IL-3 for 30 minutes. In agreement with our reported data [20], expression of the STAT5 target genes *Cis*, *Osm* and *c-Myc* was inhibited by SFN in a dose-dependent manner, while expression of the housekeeping gene *36b4* remained unaffected (Fig 2A). Interestingly, expression of *Cis*, *Osm* and *c-Myc* was similarly inhibited by GMG-ITC in a dose-dependent manner (Fig 2A). In both cases, significant inhibition was already observed at 0.4 μM ITC (15–30% inhibition depending on the target gene). The inhibition level on STAT5 target gene expression in the presence of GMG-ITC and SFN was comparable at 0.4 and 2 μM, while inhibition in the presence of 10 μM GMG-ITC was slightly stronger than that observed with the same concentration of SFN (100% vs. 77–93% respectively) (Fig 2A). Cells were subjected to trypan blue exclusion assay after 3 hours and 21 hours of ITC treatment to follow ITC-induced cell viability. As previously observed [20], only treatment with higher concentrations (10 μM) of SFN led to growth inhibition and partial (34%) cell death after 21 hours (Fig 2B). In comparison, GMG-ITC significantly inhibited growth at a concentration of 2 μM after 21 hours of treatment (Fig 2B). Moreover, 10 μM GMG-ITC induced 43% cell death already after 3 hours treatment and 100% cell death at 21 hours (Fig 2B). Therefore, while both SFN and GMG-ITC exert a similar inhibitory activity toward IL-3-mediated expression of STAT5 target genes, GMG-ITC appears to exhibit a higher cytotoxicity than SFN on stimulated Ba/F3 cells. This was confirmed by using a WST-1 assay, measuring changes in the metabolic activity of Ba/F3 cells upon treatment with 0.1–100 μM SFN or with 0.02–20 μM GMG-ITC (Fig 2C). Low (20%) cytotoxicity was reached following 2.5 hours treatment with 10 μM SFN or 2 μM GMG-ITC. However, toxicity increased to 60% in Ba/F3 cells treated with 100 μM SFN or 20 μM GMG-ITC (Fig 2C). Together with the massive cell death associated with prolonged treatment of Ba/F3 cells with 10 μM GMG-ITC, these data indicate that GMG-ITC is toxic for Ba/F3 cells at concentrations above 2 μM.

### Inhibition of STAT5 target gene expression by moringin occurs downstream of STAT5 phosphorylation

We showed before that SFN inhibits expression of STAT5 target genes without interfering with STAT5 activation by phosphorylation [20]. We therefore investigated whether treatment of Ba/F3 cells with GMG-ITC, at a concentration impairing induction of STAT5 target gene expression without causing toxicity (2 μM), had an effect on STAT5 phosphorylation. Ba/F3 cells were pre-treated 30 minutes with 10 μM SFN or 2 μM GMG-ITC prior to IL-3 stimulation. STAT5 phosphorylation was evaluated by Western blot using antibodies specific for phospho-STAT5 (pSTAT5). STAT5 protein levels were verified using antibodies recognizing STAT5A and STAT5B proteins (STAT5). Equal protein loading was confirmed by probing the blot with antibodies directed against α-tubulin. As formerly reported [20], SFN treatment did not alter IL-3-induced STAT5 phosphorylation (Fig 2D). Similarly, treatment with GMG-ITC had neither an apparent effect on the level of STAT5 phosphorylation, nor on its protein level (Fig 2D). These observations suggest that GMG-ITC inhibits STAT5 activity at a step downstream of STAT5 activation, as previously proposed for SFN [20].

### Moringin and sulforaphane inhibit IFNα-mediated induction of interferon-stimulated genes without altering STAT1 and STAT2 phosphorylation

**Fig 3:**
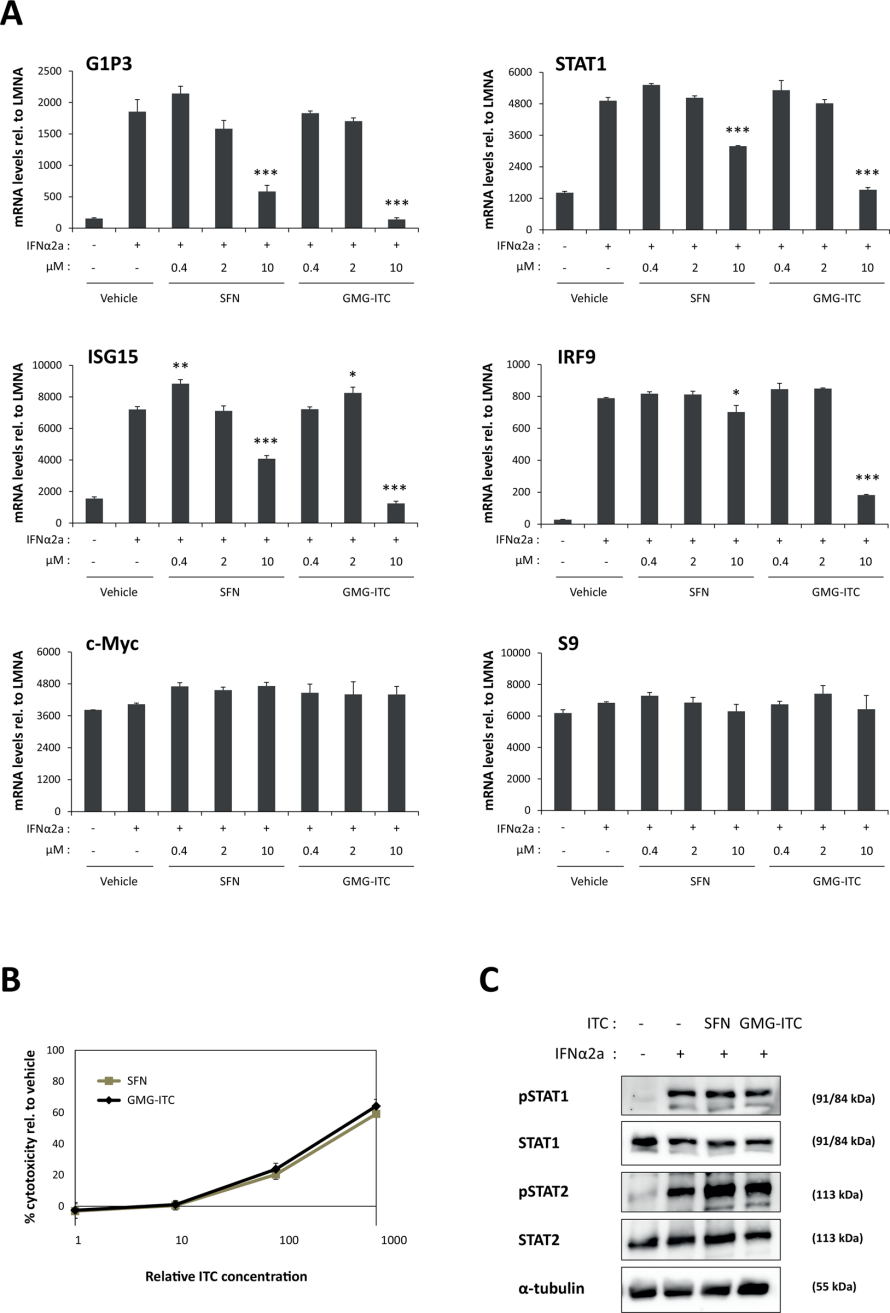
SFN and GMG-ITC inhibit IFNα-mediated induction of STAT1/STAT2 target genes in a dose-dependent manner in HeLa cells. **(A)** HeLa cells were treated 30 minutes with 0.01% DMSO (vehicle) or with 0.4, 2 and 10 μM SFN or GMG-ITC, and further stimulated with IFNα2a for 4 hours. Expression of interferon-stimulated genes (*G1P3*, *ISG15*, *STAT1*, *IRF9*), of an interferon-independent gene (*c-Myc*) and of the housekeeping gene *S9* was analyzed by quantitative RT-PCR. One-way ANOVA with Dunnett’s post test was used to assess differences between isothiocyanate-treated conditions vs. the vehicle-treated IFNα2a-stimulated control; *P<0.05, **P<0.01, ***P<0.001. **(B)** GMG-ITC exhibits a higher toxicity than SFN in HeLa cells. HeLa cells were pre-treated 30 minutes with 0.1, 1, 10 and 100 μM SFN or with 0.02, 0.2, 2 and 20 μM GMG-ITC (0.1% DMSO in all conditions) prior to addition of the WST-1 reagent. Assay was conducted for 4 hours, as described in Materials and Methods, and toxicity was normalized to the DMSO vehicle control. The x-axis indicates relative isothiocyanate (ITC) concentrations, with 1 corresponding to 0.1 μM SFN and 0.02 μM GMG-ITC. **(C)** SFN and GMG-ITC do not alter IFNα-induced phosphorylation of STAT1 and STAT2. HeLa cells were pre-treated 30 minutes with 10 μM SFN, 2 μM GMG-ITC or 0.01% DMSO as vehicle control (-) and further stimulated 30 minutes with IFNα2a. Whole-cell protein lysates were analysed by Western blot using antibodies directed against pSTAT1, pSTAT2, STAT1, STAT2 and α-tubulin (loading control). ITC, isothiocyanate.

The finding that SFN and GMG-ITC both inhibit STAT5-mediated transcription raises the question of their effect on the activity of other STAT family members. SFN was reported to inhibit IL-6-induced as well as constitutive STAT3 activity in a number of cancer cell lines [70–72]. One publication described the ability of SFN to inhibit interferon (IFN)γ-mediated STAT1 activation [73]. To the best of our knowledge, nothing is known about the effect of SFN and GMG-ITC on IFNα signaling. Upon stimulation with IFNα, STAT1 and STAT2 are phosphorylated and associate with IRF9 to form the so-called ISGF3 complex. In turn, ISGF3 activates expression of interferon-stimulated genes. To investigate the effect of SFN and GMG-ITC on STAT1/STAT2-mediated transcription, HeLa cells were pre-treated 30 minutes with 0.4–10 μM SFN or GMG-ITC and stimulated with IFNα2a for 4 hours. Expression of the interferon-stimulated genes (ISGs) *G1P3*, *ISG15*, *STAT1* and *IRF9* and of the IFN-independent genes *c-Myc* and *S9* was analysed by RT-qPCR (Fig 3A). At the concentrations of 0.4 and 2 μM, SFN and GMG-ITC did not inhibit IFNα-induced expression of ISGs. At 10 μM however, ISG induction was strongly inhibited by GMG-ITC (80–100% inhibition) and partially inhibited by SFN (11–75% inhibition). Induction of the four ISGs investigated was differentially affected by ITC treatment, *IRF9* being the least affected (11% and 80% inhibition by 10 μM SFN and GMG-ITC respectively) and *G1P3* being more strongly inhibited (75% and 100% inhibition by 10 μM SFN and GMG-ITC respectively). Interestingly, basal expression of *c-Myc* remained unaffected upon treatment with up to 10 μM SFN or GMG-ITC (Fig 3A), indicating that both ITCs inhibit STAT5-induced (Fig 2A) but not basal *c-Myc* transcription. Expression of the housekeeping gene *S9* was comparable in all conditions (Fig 3A).

Cellular toxicity exerted by SFN and GMG-ITC on HeLa cells was assessed as before following 4.5 hours ITC treatment. Changes in metabolic activity were comparable as those observed in Ba/F3 cells, with low (20%) cytotoxicity in the presence of 10 μM SFN or 2 μM GMG-ITC and increased (60%) cytotoxicity at 100 μM SFN or 20 μM GMG-ITC (Fig 3B). Therefore, similarly to its effect on Ba/F3 cells, GMG-ITC exerts a stronger cytotoxicity on HeLa cells than SFN. These data also suggest that SFN-mediated inhibition of STAT1/STAT2-induced transcription observed at 10 μM occurs independently of cytotoxicity, while GMG-ITC-mediated inhibition at 10 μM might result, at least in part, from cell toxicity.

We next investigated whether treatment with SFN or GMG-ITC had an effect on STAT1 and/or STAT2 phosphorylation, using non-toxic ITC concentrations. HeLa cells were pre-treated for 30 minutes with 10 μM SFN or 2 μM GMG-ITC and stimulated with IFNα2a for 30 minutes. STAT1 and STAT2 phosphorylation was evaluated by Western blot using phospho-specific antibodies. Treatment of IFNα-stimulated HeLa cells with SFN or GMG-ITC had no major effect on STAT1 and STAT2 phosphorylation and did not affect their respective protein levels (Fig 3C). Given that 10 μM SFN was sufficient to inhibit IFNα-induced ISG expression, these data indicate that SFN-mediated repression of IFNα signaling does not involve the inhibition of STAT1 or STAT2 phosphorylation. It therefore suggests a mechanism of inhibition similar to that mediated by SFN on STAT5 signaling, likely involving a downstream effect. The absence of effect of GMG-ITC on STAT1/STAT2 phosphorylation also suggests a similar mechanism. However, since 2 μM GMG-ITC had no effect on IFNα-induced ISG expression, we cannot definitely conclude that it is the case.

### Moringin and sulforaphane strongly inhibit TNF-induced expression of NF-κB target genes

**Fig 4:**
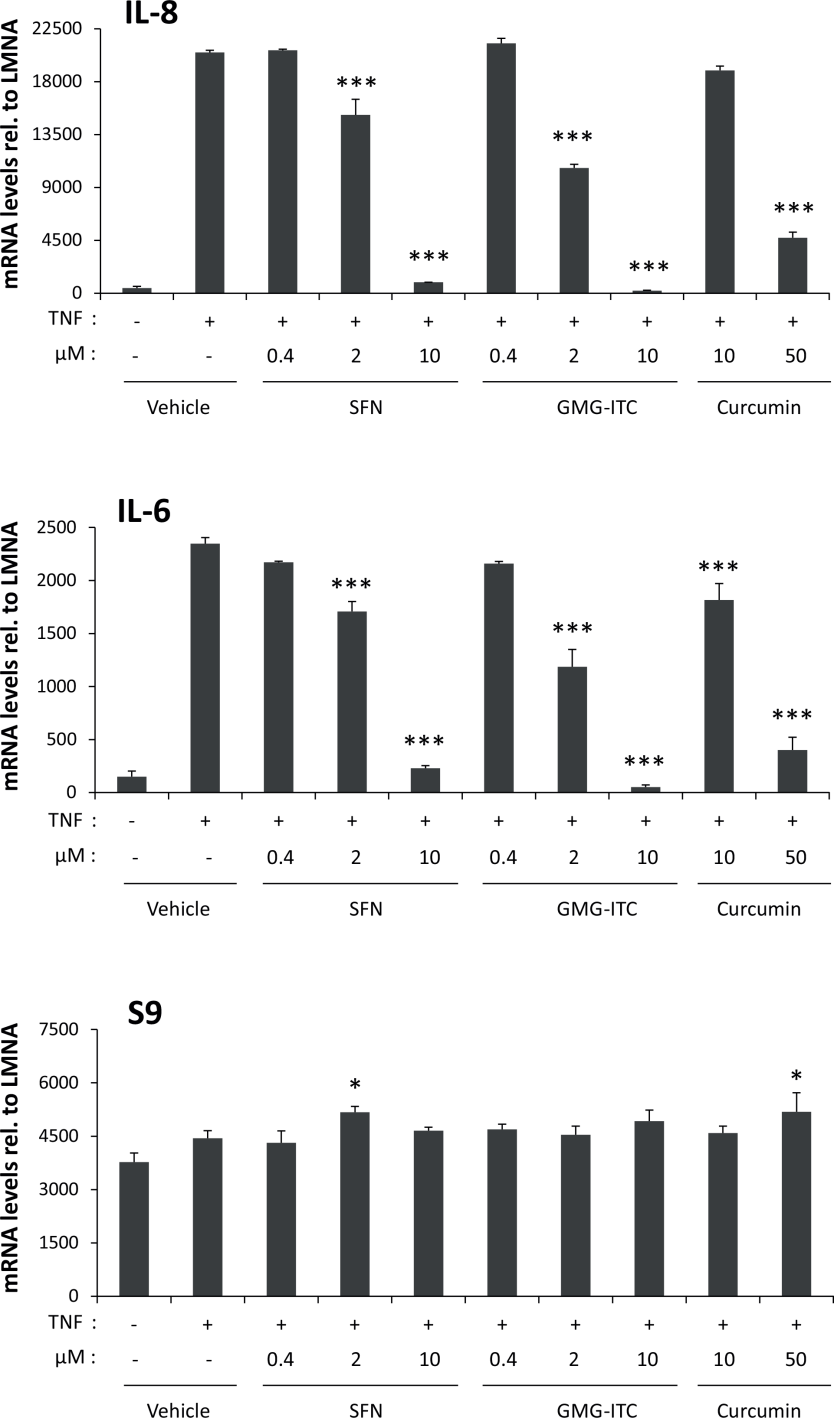
SFN and GMG-ITC inhibit TNF-mediated induction of NF-κB target genes in a dose-dependent manner in HeLa cells. HeLa cells were pre-treated 30 minutes with 0.05% DMSO (vehicle), with 0.4, 2 and 10 μM SFN or GMG-ITC or with 10 and 50 μM of the natural NF-κB inhibitor curcumin. Following drug treatment, cells were stimulated with TNF for 1 hour, and expression of the NF-κB target genes (*IL-6*, *IL-8*) and of the housekeeping gene *S9* was analyzed by quantitative RT-PCR. One-way ANOVA with Dunnett’s post test was used to assess differences between drug-treated conditions vs. the vehicle-treated TNF-stimulated control; *P<0.05, ***P<0.001.

A number of independent reports indicate that both SFN and GMG-ITC inhibit NF-κB signaling *in vitro* and *in vivo* [13,15,16,18,57,61,62,74]. One study compared the ability of SFN and GMG-ITC to inhibit constitutive NF-κB activity in cancer cell lines [57]. However, the respective inhibitory activity of SFN and GMG-ITC on a model of TNF-induced NF-κB activation has not been studied. We compared the potency of SFN and GMG-ITC to inhibit NF-κB activity, in parallel to curcumin, another natural product and acknowledged NF-κB inhibitor [75]. HeLa cells were pre-treated 30 minutes with 0.4–10 μM ITCs or with 10–50 μM curcumin and further stimulated with TNF for 1 hour. Expression of NF-κB target genes (*IL-8*, *IL-6*) and of the housekeeping gene *S9* was assessed by RT-qPCR (Fig 4). TNF-induced expression of *IL-8* and *IL-6* was inhibited by all three compounds in a dose-dependent manner. Significant inhibition by SFN and GMG-ITC was detected already at 2 μM and was stronger in the presence of GMG-ITC (~50% inhibition compared to ~28% with SFN). Complete inhibition of NF-κB target gene expression was observed at 10 μM SFN or GMG-ITC, while inhibition only reached 8–24% (depending on the target gene) upon treatment with the same concentration of curcumin. Expression of the control gene *S9* remained mostly unchanged in all conditions. Together, these experiments reveal that both SFN and GMG-ITC inhibit NF-κB signaling with a greater potency than that mediated by the well-characterized NF-κB inhibitor curcumin. Beside, GMG-ITC appears to be more potent at 2 μM than SFN in inhibiting TNF-induced gene expression.

### Sulforaphane acts as a chemopreventive agent in a model of STAT5-induced cell transformation

**Fig 5:**
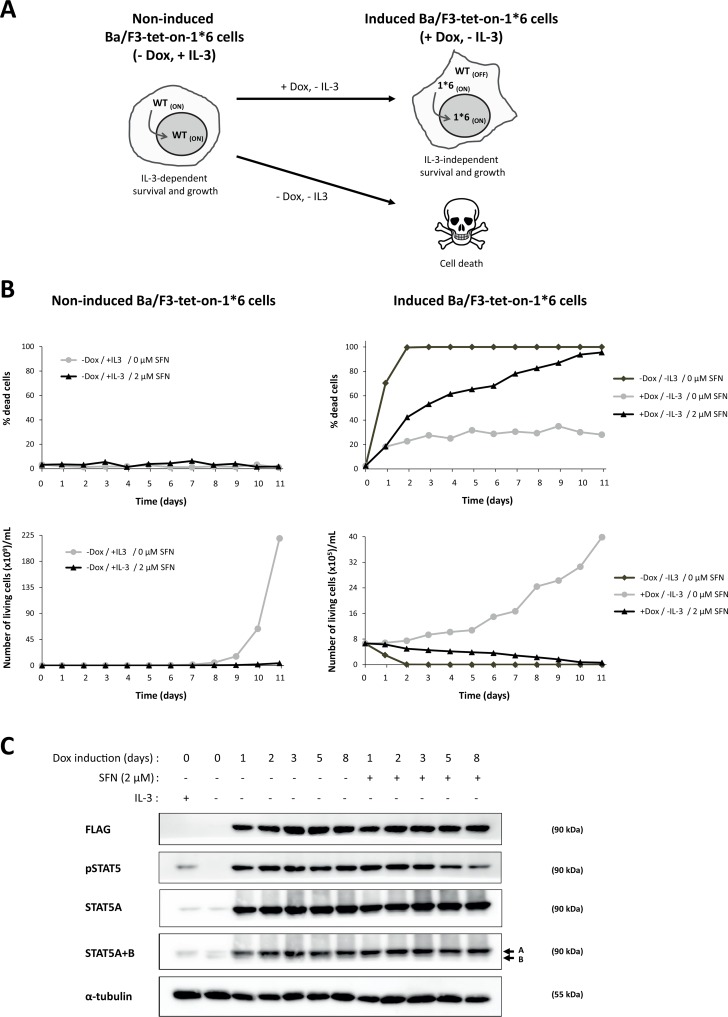
SFN treatment impairs STAT5A-1*6-mediated survival and growth of Ba/F3 cells. **(A)** Schematic representation of the growth and survival behavior of non-induced and doxycycline (Dox)-induced Ba/F3-tet-on-1*6 in the presence or absence of IL-3. Non-induced Ba/F3-tet-on-1*6 cells are IL-3-dependent (-Dox, +IL-3), like parental Ba/F3 cells. IL-3-growing cells express active endogenous STAT5 (WT_ON_). Upon IL-3 withdrawal, endogenous STAT5 activity is turned off (WT_OFF_) and non-induced Ba/F3-tet-on-1*6 cells rapidly undergo apoptosis (-Dox, -IL-3). Treatment of Ba/F3-tet-on-1*6 cells with doxycycline results in the expression of the constitutively active STAT5A-1*6 mutant (1*6_ON_) [76], allowing cells to survive and eventually grow in the absence of IL-3 (+Dox, -IL-3). In these conditions, endogenous wild-type STAT5 is inactive (WT_OFF_). **(B, C)** Non-induced and Dox-induced Ba/F3-tet-on-1*6 cells were grown in the absence or presence of IL-3, and were treated with 2 μM SFN or left untreated, as indicated, for up to 11 days. Living and dead cells were counted every day by trypan blue exclusion **(B)** and were harvested for Western blot analysis **(C)**. The ‘no Dox, no IL-3’ control cells shown in lane 2 of the Western blot were harvested after 6 hours of IL-3 withdrawal. The FLAG-specific antibody recognizes flag-tagged STAT5A-1*6 in Dox-induced cells; pSTAT5 detects phosphorylated STAT5 (endogenous WT in IL-3-growing non-induced cells and STAT5A-1*6 in Dox-induced cells); STAT5A-specific antibodies (STAT5A and STAT5A+B) recognize both endogenous WT STAT5A and induced STAT5A-1*6; STAT5B-specific antibodies (STAT5A+B) recognize endogenous WT STAT5B proteins; α-tubulin was used as a loading control. Western blot analysis showed that STAT5A-1*6 was overexpressed compared to endogenous STAT5A and STAT5B upon treatment with doxycycline. Moreover, expression of STAT5A-1*6 remained constant over time regardless of the presence of SFN. By contrast, STAT5A-1*6 phosphorylation decreased after 5 days of treatment with 2 μM SFN.

The ability of natural compounds such as the ITCs SFN and GMG-ITC to inhibit multiple signaling pathways controlling cell proliferation, cell survival, immune modulation and inflammation, make them attractive agents in particular for cancer prevention. To assess the chemopreventive activity of natural compounds, we developed a cellular model of STAT5-induced oncogenic transformation, so-called Ba/F3-tet-on-1*6 (Fig 5A). Ba/F3-tet-on-1*6 cells express a constitutively active mutant of STAT5 (STAT5A-1*6) upon treatment with the tetracycline analog doxycycline [54,76]. Upon expression of STAT5A-1*6, Ba/F3 cells—which are normally dependent on IL-3 for survival and growth—acquire the ability to survive in the absence of IL-3, eventually grow in an IL-3-independent manner and are highly tumorigenic *in vivo* [54,68,76,77]. Ba/F3-tet-on-1*6 cells thus represent an ideal experimental system to investigate the potential chemopreventive activity of natural compounds. SFN was chosen for these experiments due to its low toxicity in long-term cultures. Expression of STAT5A-1*6 was induced by doxycycline treatment of Ba/F3-tet-on-1*6 cells, in the absence or presence of IL-3 and of increasing concentrations of SFN (0, 1 and 2 μM). SFN treatment of non-induced cells grown in the presence of IL-3 (non-transformed control cells) resulted in a dose-dependent cell growth inhibition but did not induce cell death (S1 Fig and Fig 5B). As predicted, non-induced cells withdrawn from IL-3 died within the first two days (S1 Fig and Fig 5B). Seventy to 80% of Ba/F3-tet-on-1*6 cells expressing constitutive active STAT5A-1*6 survived in IL-3-free culture and started growing after 5–6 days in culture (S1 Fig and Fig 5B). Interestingly, induced cells grown in the presence of SFN showed reduced survival and proliferation in a dose-dependent manner (S1 Fig and Fig 5B). Treatment of STAT5A-1*6-transformed cells with 2 μM SFN resulted in 100% cell death after 6–11 days in culture (S1 Fig and Fig 5B). These data show that SFN is able to reverse the survival and growth advantage exerted by STAT5A-1*6 and to induce the death of transformed cells.

Doxycycline-dependent expression and phosphorylation of FLAG-tagged STAT5A-1*6 was verified by Western blot using FLAG-, STAT5- and phospho-STAT5-specific antibodies (Fig 5C). STAT5A-1*6 transgene was constantly expressed over the course of the experiment (FLAG and STAT5 blots). STAT5A-1*6 phosphorylation level was equal in untreated induced cells. However, the level of phosphorylated STAT5 decreased at days 5 and 8 in cells treated with 2 μM SFN (Fig 5C). Whether this reduction is a consequence of the high cell death associated with these samples (65% and 83% cell death at day 5 and day 8 respectively) or reflects a downregulation of STAT5A-1*6 activity by SFN remains to be shown.

## Discussion

This study aimed to analyse and compare the inhibitory activities of two natural ITCs, sulforaphane (SFN) and moringin (GMG-ITC). We focused on a number of signaling pathways essential for the control of cell proliferation, apoptosis, immune response and inflammation and known to be dysregulated in various chronic diseases. Gene expression analyses revealed that SFN and GMG-ITC differentially affected the investigated signaling pathways. We found that both SFN and GMG-ITC inhibit IL-3-induced STAT5 signaling at concentrations as low as 0.4 μM. This is to our knowledge the first demonstration of an inhibitory effect of GMG-ITC on STAT5-mediated transcription. We also showed for the first time that both ITCs inhibit to some extent IFNα-induced STAT1 and STAT2 activity, although the effective concentration was 25-times higher (10 μM) than that required for inhibition of STAT5 activity. In both cases, inhibition of JAK/STAT signaling did not take place at the level of STAT protein phosphorylation, suggesting a downstream inhibitory event. Interestingly, SFN and GMG-ITC specifically impaired STAT5-dependent (Ba/F3) but not basal (HeLa) c-Myc expression, highlighting the specificity of action of these ITCs. Furthermore, SFN and GMG-ITC inhibit TNF-induced NF-κB signaling, thus supporting previous studies performed in other experimental systems [57,61–63]. Inhibition of NF-κB activation by ITCs was detectable at concentrations of 2 μM. SFN and GMG-ITC are thus more potent inhibitors of NF-κB than the acknowledged inhibitor curcumin, which required a concentration of at least 10 μM to mediate a comparable inhibitory effect on NF-κB target gene expression. While SFN and GMG-ITC were equally potent inhibitors of STAT5 signaling, GMG-ITC appeared to be more effective in the inhibition of STAT1/STAT2 and of NF-κB than SFN. On the other hand, GMG-ITC treatment was more cytotoxic than SFN treatment for both Ba/F3 and HeLa cells. Therefore, one cannot exclude that the stronger inhibitory activity observed upon treatment with 10 μM GMG-ITC is related to its cytotoxicity. In particular, this might be the case for the inhibition of IFNα-induced STAT1/STAT2/IRF9 signaling which was only evident at higher ITC concentrations. Interestingly, the IFN-regulated gene IRF9 showed a drastic difference in sensitivity to SFN and GMG-ITC treatment, being only marginally affected upon treatment with 10 μM SFN and strongly inhibited by GMG-ITC at the same concentration. Since IRF9 is an essential cofactor of the ISGF3 complex, a stronger inhibition of IRF9 by GMG-ITC might also explain the overall stronger inhibition of ISG expression by GMG-ITC.

The observation that STAT5 target gene expression is similarly inhibited by SFN and GMG-ITC treatment may suggest a similar inhibitory mechanism. This possibility is supported by the observation that neither SFN nor GMG-ITC treatment interfere with STAT5 phosphorylation. We showed before that SFN treatment does not affect STAT5 activation (phosphorylation), nor its binding to DNA, and only marginally interferes with STAT5-mediated recruitment of RNA polymerase II [20]. Furthermore, SFN treatment had no apparent effect on histone acetylation levels in Ba/F3 cells [20], suggesting that it does not act as a histone deacetylase inhibitor [78,79]. Our data therefore supported a model in which SFN targets STAT5 transcriptional activity at a step following binding of activated STAT5 to DNA and recruitment of the transcriptional machinery. Whether inhibition by GMG-ITC involves a similar mechanism remains to be shown.

The finding that SFN and GMG-ITC treatment does not alter STAT1 and STAT2 phosphorylation similarly suggest that inhibition of IFN-stimulated gene expression takes place at the transcriptional level. This proposition is further supported by the fact that expression of the ISGs investigated (*G1P3*, *ISG15*, *STAT1* and *IRF9*) was differentially affected upon SFN and GMG-ITC treatment, in favor of a promoter-specific regulation. Further investigations will be necessary to address the level of inhibition by these natural ITCs. It should be noted that, in contrast to STAT1/2/5 proteins, constitutive and IL-6-induced STAT3 phosphorylation was shown to be reduced upon treatment by SFN in a number of cancer cell lines [70–72], thus revealing multiple modes of regulation of STAT proteins by ITCs.

In contrast to STAT5 target gene expression, which was equally affected by SFN and GMG-ITC in Ba/F3 cells, TNF-induced NF-κB-mediated transcription in HeLa cells was more strongly inhibited by GMG-ITC than by SFN. Similarly, GMG-ITC was more effective than SFN in inhibiting NF-κB activity in macrophages and multiple myeloma cells [57]. This difference in activity might reflect a distinct mode of action or a differential ability of GMG-ITC and SFN to interact with common target proteins, possibly due to differences in their chemical structure (Fig 1). While little is known about the mechanism of inhibition of NF-κB signaling by GMG-ITC [57,61–63,80], SFN appears to directly and indirectly inhibit NF-κB signaling at multiple levels, targeting both upstream and downstream components of the pathway [11–19].

The molecular mechanism by which SFN and GMG-ITC inhibit STAT and NF-κB signaling pathways remains to be uncovered. ITCs are electrophiles known for their ability to react with thiols [10]. SFN was shown to react with free sulfhydryl groups of cysteines in proteins and modify their function [8,16,81,82]. Therefore, it is likely that SFN and GMG-ITC react with cysteine residues within STAT proteins or with a cofactor of STAT-mediated transcription, as well as with components of the NF-κB signaling pathway. On the other hand, SFN reacts with intracellular glutathione (GSH), which contributes to the rapid cellular uptake and accumulation of SFN and to a concomitant drop in endogenous GSH levels [9]. A drop in GSH might trigger S-glutathionylation of cysteines within STAT and/or NF-κB transcription factors, thereby inhibiting their activity, as demonstrated for other electrophilic natural compounds [83–86]. Although thiol- and redox-dependent mechanisms of inhibition of NF-κB activity by SFN have been reported [16–19], further investigations will be necessary to determine their contribution in SFN- and GMG-ITC-mediated inhibition of STAT and NF-κB signaling pathways.

Natural ITCs such as SFN and GMG-ITC have long been known for their chemopreventive activities against various pathologies. Using our STAT5-induced cell transformation assay, we provide strong evidence that SFN can counteract STAT5A-1*6-driven oncogenesis, by inducing cell death of transformed—but not of healthy—cells. The observation that STAT5A-1*6 phosphorylation was reduced in SFN-treated cells suggests that inhibition of STAT5 activity contributes at least in part to SFN-induced cell death. However, given the pleiotropic activities of SFN, further experiments will be required to identify the critical targets of SFN in Ba/F3 cells transformed by constitutive active STAT5.

In conclusion, our data indicate that SFN and GMG-ITC are potent inhibitors of STAT5, NF-κB and, to a lesser extent, STAT1/STAT2 signaling pathways. Given the implication of these pathways in chronic pathologies such as cancer, inflammatory diseases and immune disorders, the dietary and easily available ITCs SFN and GMG-ITC represent attractive and valuable chemopreventive agents.

## Supporting Information

S1 Fig SFN treatment prevents STAT5A-1*6-mediated survival and growth of Ba/F3 cells in a dose-dependent manner. Non-induced and Dox-induced Ba/F3-tet-on-1*6 cells were grown in the absence or presence of IL-3, and were treated with 0, 1 or 2 μM SFN, as indicated, for up to 8 days. Living and dead cells were counted every day by trypan blue exclusion. Data are expressed as percentage of dead cells (% dead cells) and as density of living cells (number of living cells/mL). To better visualize SFN-induced cell growth inhibition in non-induced cells, the Y-axis scale of the corresponding graph was expanded (lower left graph).
(EPS)

S1 File Raw data. RT-qPCR CT values; WST-1 OD values; uncropped Western blots.
(PDF)
