# Supplementary material for: The Chemopreventive Phytochemical Moringin Isolated from Moringa oleifera Seeds Inhibits JAK/STAT Signaling
Source: PLoS One. 2016 Jun 15;11(6):e0157430. doi: 10.1371/journal.pone.0157430 (PMC4909285; doi:10.1371/journal.pone.0157430)

Michl et al. - Supporting File S1 (Raw data)

1. RT-qPCR assays - CT values

Figure 2A - CT values (Ba/F3 cells)

	#1	#2	#3	#4	#5	#6	#7	#8
	- IL-3	+ IL-3						
	DMSO	DMSO	SFN			GMG-ITC		
	0.01%	0.01%	0.4 µM	2 µM	10 µM	0.4 µM	2 µM	10 µM
**Cis**	28.57	23.42	23.75	24.3	25.65	23.96	24.17	28.57
**Cis**	28.63	23.47	23.71	24.21	25.46	23.86	24.01	28.5
**Osm**	28.79	23.58	24.15	24.76	26.7	24.71	25.11	29.65
**Osm**	27.72	23.57	24.21	*25.14	27.52	24.37	25.31	29.94
**Osm**	28.05	23.71	*23.67	24.74	26.84	24.37	25.49	**
**Osm**	29.57	23.83	*23.61	24.44	27.48	24.4	25.23	29.76
**c-Myc**	25.15	22.57	23.11	23.22	24.89	22.78	23.55	25.84
**c-Myc**	25.2	22.49	23.08	23.39	24.63	23.01	23.27	26
**c-Myc**	25.61	*22.18	22.88	23.12	24.96	23.02	23.72	25.51
**c-Myc**	25.81	22.4	23	23.44	24.83	22.85	23.5	25.64
**c-Myc**	25.62	22.39	23	23.49	24.9	22.9	23.31	25.33
**36b4**	16.92	16.98	16.9	17	16.95	17.21	17.1	17.24
**36b4**	16.9	17.08	16.9	17.02	17.12	17.24	17.17	17.39
**S9**	17.84	17.94	18.1	*18.27	18.16	18.29	18.07	18.19
**S9**	18.07	18.06	18.02	18.01	18.12	18.27	18.02	17.99
**S9**	17.9	18.02	17.99	*18.33	18.07	18.19	18.13	18.32
**S9**	17.79	18.03	18.25	17.9	18.19	18.3	17.95	18.32
**S9**	18.02	18.03	18.07	18.1	18.16	18.24	17.93	18.19

* outlier (excluded from average calculation)
** no amplification (excluded from average calculation)

Figure 3A - CT values (HeLa cells)

	#1	#2	#3	#4	#5	#6	#7	#8
	-IFNα2a	+IFNα2a						
	DMSO	DMSO	SFN			GMG-ITC		
	0.01%	0.01%	0.4 µM	2 µM	10 µM	0.4 µM	2 µM	10 µM
**G1P3**	26.57	22.6	22.4	22.83	24.62	22.67	22.89	27.04
**G1P3**	26.41	22.81	22.29	23	24.28	22.71	22.83	26.65
**ISG15**	23.22	20.77	20.27	20.7	21.59	20.73	20.54	23.57
**ISG15**	23.09	20.72	20.33	20.79	21.69	20.69	20.63	23.79
**STAT1**	23.34	21.32	20.97	21.23	21.99	21.26	21.39	23.33
**STAT1**	23.26	21.27	20.99	21.26	22	21.06	21.33	23.45
**IRF9**	28.9	23.93	23.72	23.85	24.12	23.85	23.86	26.43
**IRF9**	29.02	23.94	23.75	23.9	24.24	23.76	23.87	26.48
**c-Myc**	21.86	21.57	21.24	21.36	21.4	21.48	21.28	21.93
**c-Myc**	21.86	21.59	21.18	21.41	21.46	21.33	21.62	21.79
**S9**	21.11	20.81	20.55	20.75	20.95	20.78	20.67	21.46
**S9**	21.19	20.83	20.61	20.85	21.08	20.84	20.81	21.18
**LMNA**	20.45	20.35	20.06	20.32	20.44	20.25	20.27	20.79
**LMNA**	20.43	20.33	20.25	20.35	20.32	20.2	20.41	20.88
**LMNA**	20.46	20.2	20.15	20.19	20.45	20.12	20.14	20.52
**LMNA**	20.55	20.24	20.19	20.2	20.34	20.27	20.25	20.51
**LMNA**	*21.02	20.25	19.96	20.26	20.18	20.34	20.34	20.51

* outlier (excluded from average calculation)

Figure 4 - CT values (HeLa cells)

	#1	#2	#3	#4	#5	#6	#7	#8	#9	#10
	-TNF	+TNF								
	DMSO	DMSO	SFN			GMG-ITC			Curcumin	
	0.05%	0.05%	0.4 µM	2 µM	10 µM	0.4 µM	2 µM	10 µM	10 µM	50 µM
**IL-8**	25.41	19.44	19.38	19.61	24.01	19.54	20.63	25.87	19.61	22.14
**IL-8**	24.68	19.46	19.37	19.79	24	19.5	20.57	26.26	19.57	21.94
**IL-6**	27.06	*22.86	*22.93	22.88	26.13	*23.27	23.94	28.4	23.07	26.15
**IL-6**	26.59	22.55	22.62	22.91	25.85	22.83	23.82	28.4	22.84	25.24
**IL-6**	26.06	22.6	22.63	22.76	26.09	22.81	23.56	27.57	23.02	25.53
**S9**	21.81	21.73	21.7	21.26	21.68	21.73	21.86	21.42	21.72	*21.73
**S9**	21.96	*22.08	21.57	*21.71	21.64	*22.22	21.89	21.64	21.62	22.01
**S9**	21.96	21.59	21.52	21.2	21.7	21.72	21.72	21.52	21.57	*21.62
**S9**	21.77	21.65	21.76	21.29	*21.41	21.65	21.85	21.53	21.64	21.8

* outlier (excluded from average calculation)

2. WST-1 assays - OD 450/620nm

Figure 2C - Ba/F3 cells

			SFN				GMG-ITC			
	Triton-X100	DMSO 0.1%	0.1 µM	1 µM	10 µM	100 µM	0.02 µM	0.2 µM	2 µM	20 µM
450-620	0.058	1.698	1.403	1.864	1.387	0.839	1.848	1.824	1.491	0.756
	0.061	2.022	1.828	1.761	1.387	0.802	1.897	1.805	1.318	0.752
	0.073	1.65	1.886	1.759	1.378	0.804	1.895	1.762	1.521	0.737
**Average**	**0.064**	**1.790**	**1.706**	**1.795**	**1.384**	**0.815**	**1.880**	**1.797**	**1.443**	**0.748**
**StDev**	**0.008**	**0.202**	**0.264**	**0.060**	**0.005**	**0.021**	**0.028**	**0.032**	**0.110**	**0.010**

Figure 3B - HeLa cells

			SFN				GMG-ITC			
	Triton-X100	DMSO 0.1%	0.1 µM	1 µM	10 µM	100 µM	0.02 µM	0.2 µM	2 µM	20 µM
450-620	0.18	1.454	1.449	1.492	1.24	0.729	1.434	1.495	1.205	0.627
	0.189	1.475	1.531	1.489	1.166	0.683	1.556	1.429	1.113	0.709
	0.182	1.501	1.566	1.426	1.23	0.719	1.536	1.462	1.188	0.602
**Average**	**0.184**	**1.477**	**1.515**	**1.469**	**1.212**	**0.710**	**1.509**	**1.462**	**1.169**	**0.646**
**StDev**	**0.005**	**0.024**	**0.060**	**0.037**	**0.040**	**0.024**	**0.065**	**0.033**	**0.049**	**0.056**

Figure 2D (Michl et al.) – uncropped Western blots

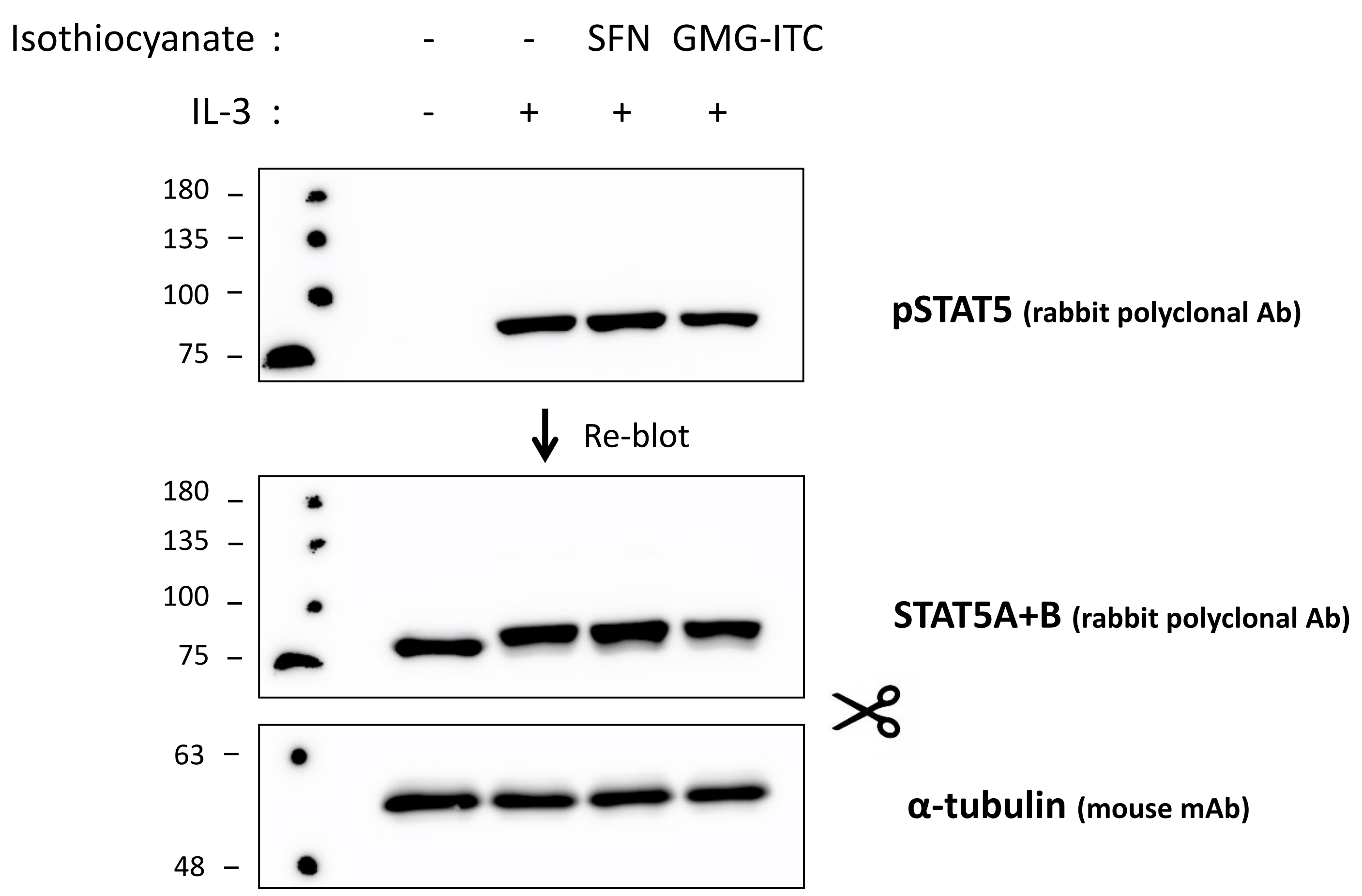

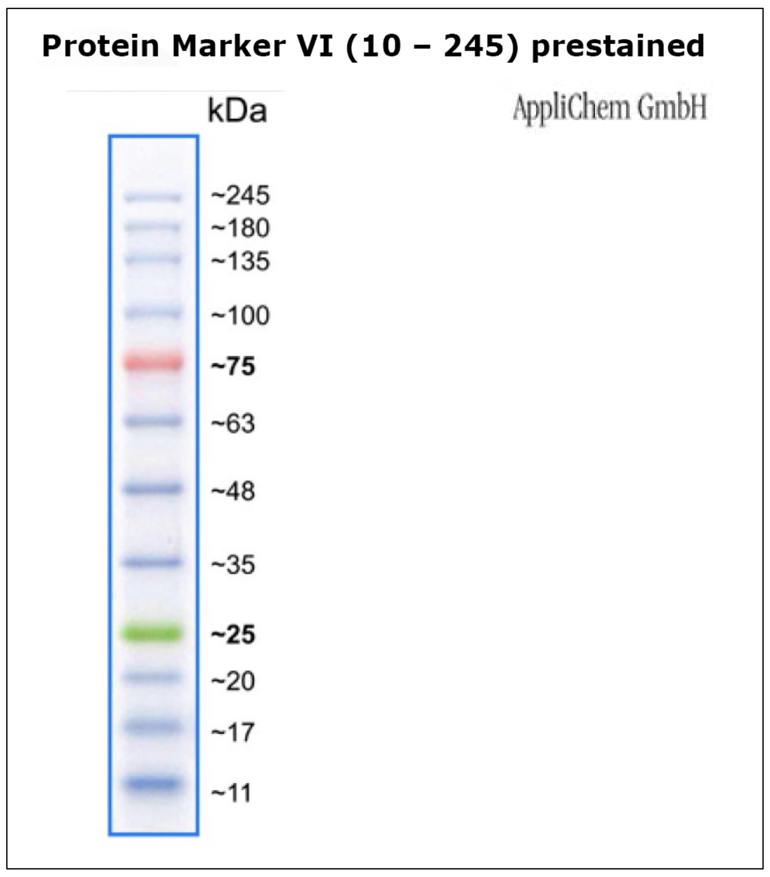

Figure 3C (Michl et al.) – uncropped Western blots

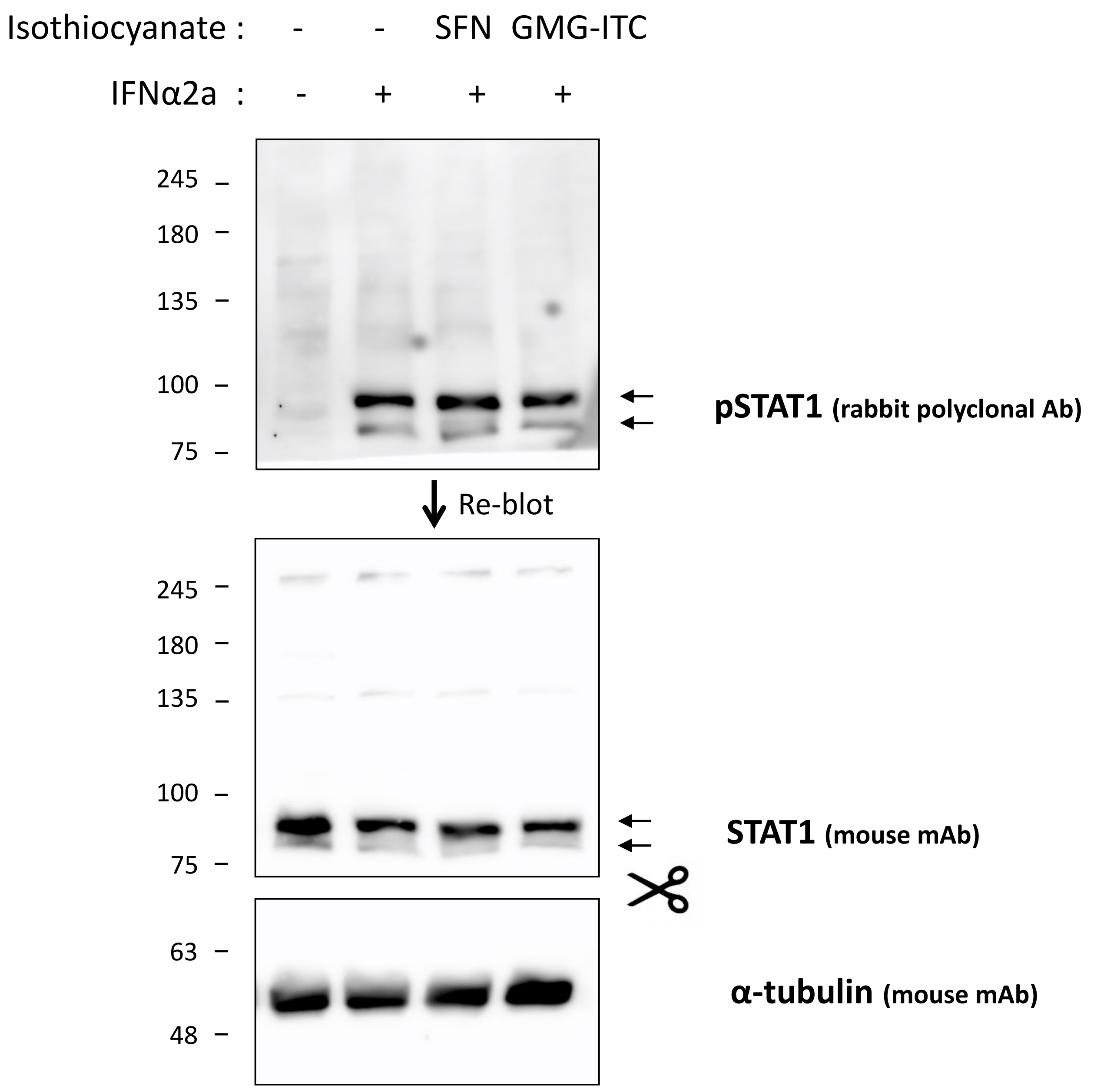

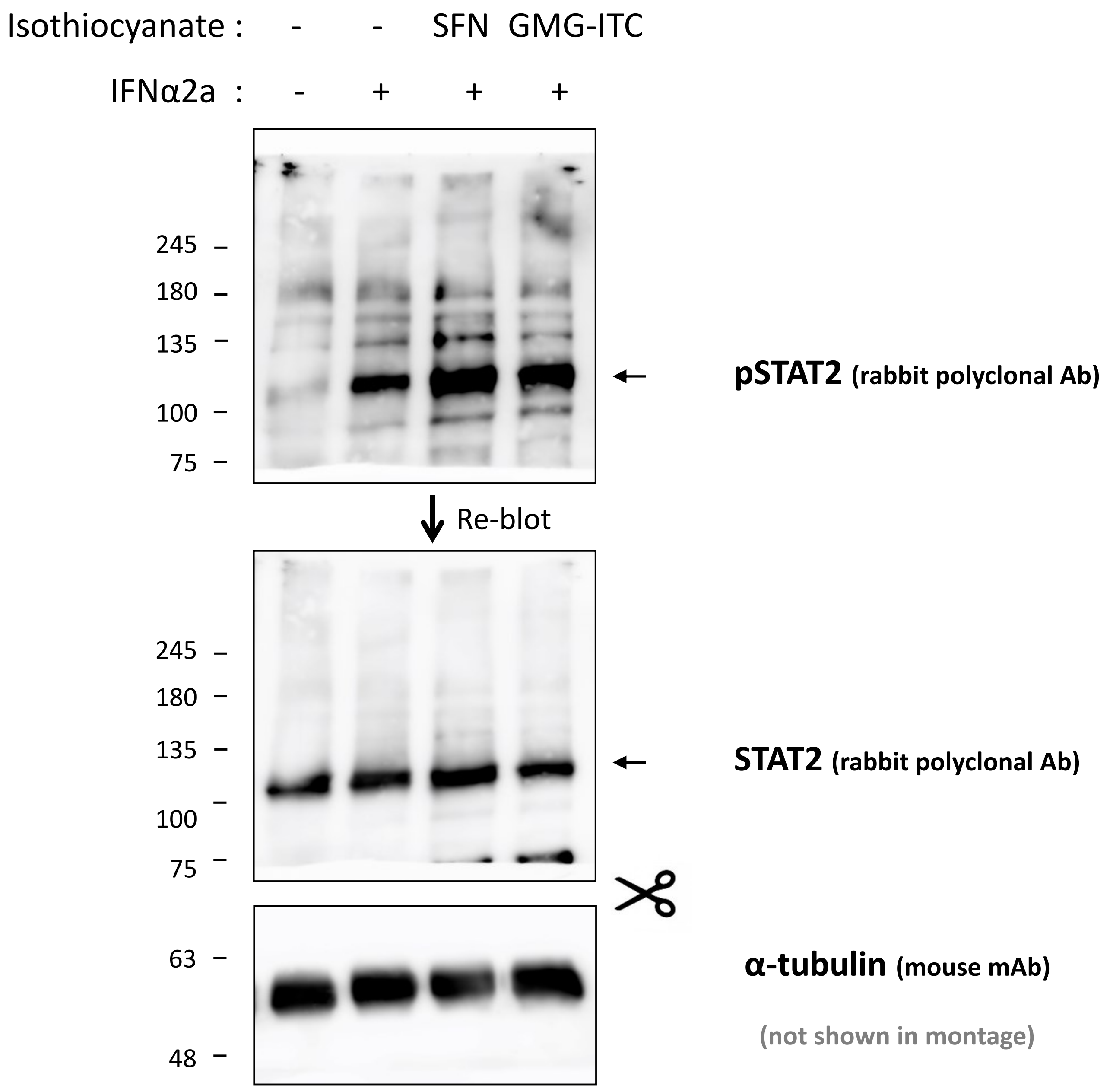

Figure 5C (Michl et al.) – uncropped Western blots

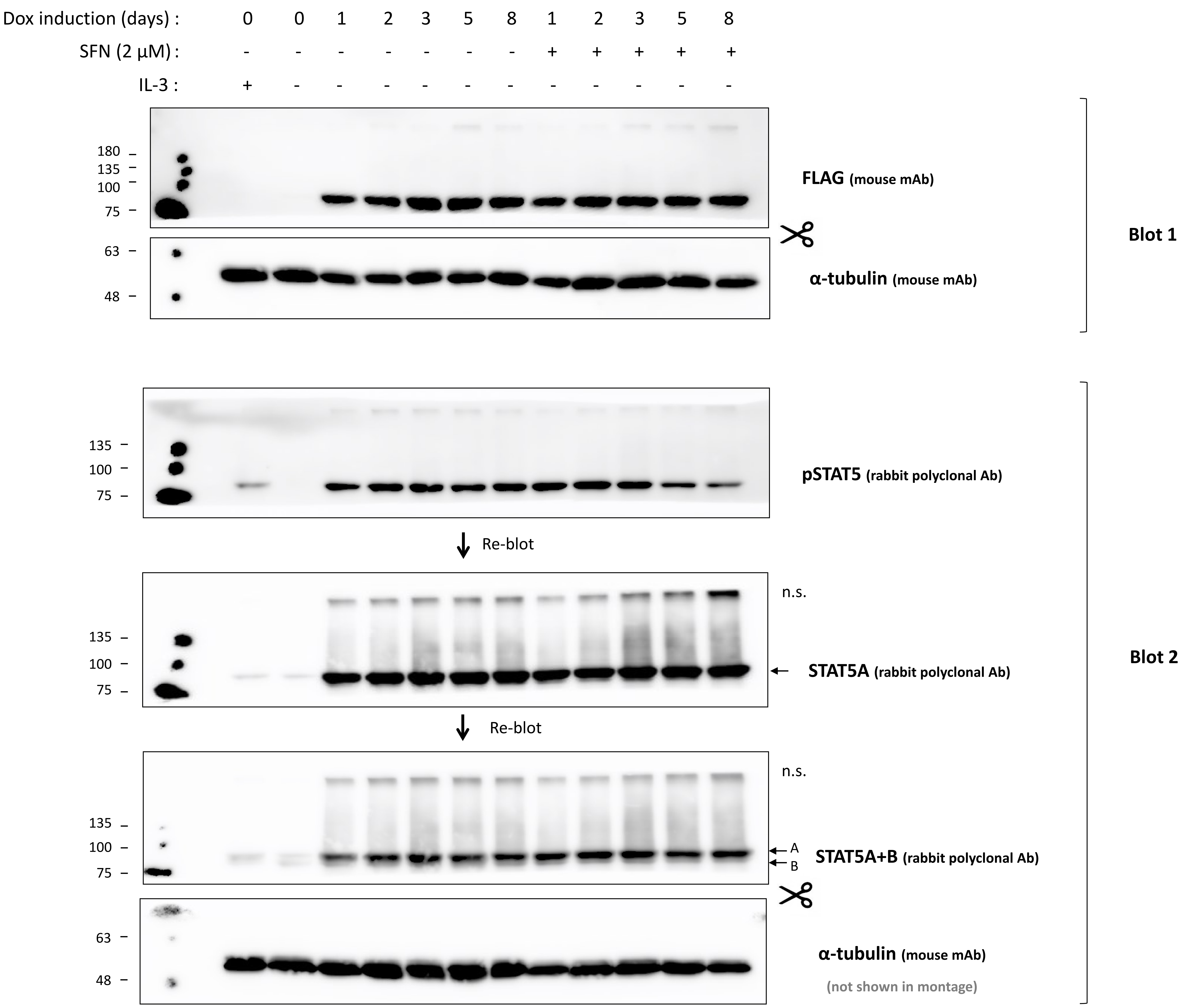

Supplement: S1 File — RT-qPCR CT values; WST-1 OD values; uncropped Western blots. (PDF) [file pone.0157430.s002.pdf]
